# NMRDyn: A Program for NMR Relaxation Studies of Protein Association

**DOI:** 10.1371/journal.pone.0003820

**Published:** 2008-11-26

**Authors:** Conan K. Wang, Horst Joachim Schirra, David J. Craik

**Affiliations:** The University of Queensland, Institute for Molecular Bioscience, Brisbane, Queensland, Australia; University of Queensland, Australia

## Abstract

Self-association is an important biological phenomenon that is associated with many cellular processes. NMR relaxation measurements provide data about protein molecular dynamics at the atomic level and are sensitive to changes induced by self-association. Thus, measurements and analysis of NMR relaxation data can provide structurally resolved information on self-association that would not be accessible otherwise. Here, we present a computer program, NMRdyn, which analyses relaxation data to provide parameters defining protein self-association. Unlike existing relaxation analysis software, NMRdyn can explicitly model the monomer-oligomer equilibrium while fitting measured relaxation data. Additionally, the program is packaged with a user-friendly interface, which is important because relaxation data can often be large and complex. NMRdyn is available from http://research1t.imb.uq.edu.au/nmr/NMRdyn.

## Introduction

Nuclear magnetic resonance (NMR) relaxation is a powerful tool for understanding the structure and dynamics of proteins. In the most common approach to the analysis of protein dynamics, relaxation data – in the form of longitudinal relaxation rates (*R_1_*), transverse relaxation rates (*R_2_*), and heteronuclear NOEs (Nuclear Overhauser Effects) – are used to derive microdynamic parameters that describe both the overall tumbling and the internal motions of a macromolecule. In the ‘model-free’ approach [1], [2], the internal dynamics are quantified using a generalized order parameter, *S^2^*, which characterizes the amplitude of motion, and an internal correlation time *τ_i_*. Additional parameters can be introduced to account for internal motions on fast and slow timescales [3]. Several computer programs have been developed to analyze relaxation data and generate the parameters that describe the rates and amplitudes of protein motions, including Modelfree [4], relax [5] and MOLDYN [6].

An important phenomenon that can affect dynamics of a protein is self-association. The formation of oligomers is a key factor in the activity of many proteins, including enzymes, ion channels, receptors and transcription factors. HIV protease is a well-known example of an enzyme that operates as a dimer [7]. Neuropeptide Y, a polypeptide hormone and neurotransmitter that is involved in the control of food intake, also forms dimers [8]. Another example is kalata B1, a member of the cyclotides [9], a novel family of circular plant peptides that act in plant defense [10], which have been shown to form tetramers in solution [11].

Nuclear magnetic resonance (NMR) relaxation measurements have the potential to provide atomic scale information about protein self-association [12], [13]. If the lifetimes of the monomer and aggregates are short on the NMR relaxation timescale, observed relaxation rates are a weighted average of the different species present [12]. Even a small amount of high molecular weight species, from a tetramer for example, can cause a significant change in the observed relaxation rates, which in an oligomerization equilibrium are strongly dependent on the protein concentration as well as the molecular volume and shape of the species being measured.

This paper describes a computer program, NMRDyn, which analyses NMR relaxation data to allow the deduction of motional parameters. Unlike existing programs, NMRdyn was designed to deal with the self-association of proteins, i.e. it is able to explicitly optimize a monomer-oligomer association model for any type of oligomer while optimizing microdynamic parameters that describe protein dynamics, and presents a user-friendly interface to facilitate the examination of data and results. NMRdyn was designed for ^15^N NMR relaxation measurements and can also deal with ^13^Cα NMR relaxation data but is not appropriate for C = O relaxation, which can be complicated by chemical shift anisotropy contributions. NMRdyn is open-source and licensed under the GNU General Public License (GPL), and the code, as well as a manual describing the use of NMRdyn, is available from http://research1t.imb.uq.edu.au/nmr/NMRdyn.

## Materials and Methods

### Theory

Nuclear spin relaxation is induced mainly by dipolar and chemical shift anisotropy interactions, which are modulated by molecular tumbling. When a protein does not self-associate, the observed relaxation data, *R_1_*, *R_2_* and *NOE*, can be expressed mathematically as shown in equations 1–3 [4].
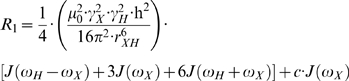
(1)

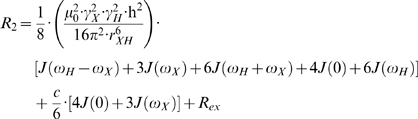
(2)

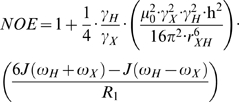
(3)Here *γ_X_* and *γ_H_* are the gyromagnetic ratios for X ( = ^13^C or ^15^N) and ^1^H respectively, *r_XH_* is the internuclear distance, and *c* represents the chemical shift anisotropy contribution. In the ‘model-free’ approach, the precise definition of the spectral density function, *J(ω)*, and the inclusion/exclusion of the chemical exchange contribution, *R_ex_*, depends on the choice of model. There are eight basic models as summarized in table 1 [1], [3], [12], [14]; models 1 and 3 use equation 4, models 2 and 4 use equation 5, models 5 and 7 use equation 6, and models 6 and 8 use equation 7. Models 1, 2, 5, and 6 differ from the other models, collectively, in that they omit the *R_ex_* term when evaluating *R_2_* according to equation 2. Equations 4 and 5 represent the original equations for spectral density derived by Lipari and Szabo [1], while equations 6 and 7 are extensions to the equations proposed by Clore *et al* [3].
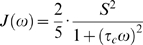
(4)

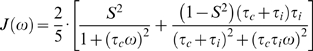
(5)

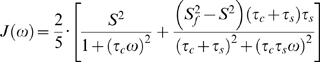
(6)

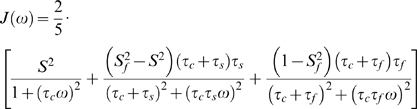
(7)Here *τ_c_* is the overall correlation time, *S^2^* is the generalized order parameter, and *τ_i_* is the internal correlation time. Potential fast internal motions are accounted for by the order parameter 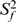 and the fast correlation time *τ_f_*.

**Table 1:** Model-free models and associated parameters.

Model	Equation	Microdynamic Parameters	*k_m_*	Explanation	Reference
*m1*	4	*τ_c_*, *S^2^*	2	very fast picosecond internal dynamics	[1]
*m2*	5	*τ_c_*, *S^2^*, *τ_i_*	3	picosecond internal dynamics	[1]
*m3*	4	*τ_c_*, *S^2^*, *R_ex_*	3	*m1* plus contributions from chemical exchange processes	[1]
*m4*	5	*τ_c_*, *S^2^*, *τ_i_*, *R_ex_*	4	*m2* plus contributions from chemical exchange processes	[1]
*m5*	6	*τ_c_*, *S^2^*, *τ_s_*, *S^2^_f_*	4	very fast picosecond and slower sub-nanosecond-nanosecond motions	[3]
*m6*	7	*τ_c_*, *S^2^*, *τ_s_*, *S^2^_f_*, *τ_f_*	5	picosecond and slower sub-nanosecond-nanosecond motions	[3]
*m7*	6	*τ_c_*, *S^2^*, *τ_s_*, *S^2^_f_*, *R_ex_*	5	*m5* plus contributions from chemical exchange processes	[3]
*m8*	7	*τ_c_*, *S^2^*, *τ_s_*, *S^2^_f_*, *τ_f_*, *R_ex_*	6	*m6* plus contributions from chemical exchange processes	[3]

When a protein does self-associate, the observed relaxation data are a weighted average of the individual relaxation data of all species present in the association equilibrium. For example, in a system containing monomers (M) and dimers (D), the observed relaxation data would be given by equations 8–10 [12], [15], [16].
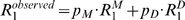
(8)

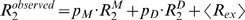
(9)

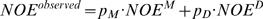
(10)where *M* and *D*, as superscripts, refer to relaxation data of the monomer and dimer, respectively, and where *p_M_* and *p_D_*, which sum to 1, are the populations of protein in the monomeric and dimeric states, respectively. The populations *p_M_* and *p_D_* can be calculated once the association constant for the dimer formation is known. 〈*R_ex_*〉 signifies contributions to *R_2_* from intramolecular exchange processes within the monomer and dimer, as well as from exchange processes between both species. If we focus on the ^15^N nucleus, contributions from monomer-dimer exchange to the R_2_ rates can occur only for those residues that differ in ^15^N chemical shift between the monomer and dimer [15]. In the case of pure monomer-dimer exchange, the 〈*R_ex_*〉 term should increase with concentration, but if a change in concentration leads to the dominance of one conformer, then the 〈*R_ex_*〉 term may decrease with increasing concentration [15]. In general, the contributions to 〈*R_ex_*〉 are not straightforward, because the timescales for conformational exchange motions and for exchange between monomer and dimer are generally similar (ms-µs). Thus, we assume 〈*R_ex_*〉 to be independent of the monomer-dimer interconversion, as has been done previously [12].

Thus, if the contributions to 〈*R_ex_*〉 are small, the general relaxation equations for a system containing different oligomeric species can be specified by equations 11–13.
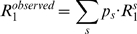
(11)

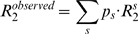
(12)

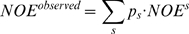
(13)where *s* iterates over all species present. The population of each species can be determined from the relevant association constant(s). If the stoichiometry of the oligomer and/or association constant is unknown, NMRdyn can extract these self-association parameters from the given relaxation data by optimizing a target function described below.

Once the form of the relaxation equations for *R_1_*, *R_2_* and *NOE* has been established, theoretical calculations can be compared to experimental measurements. In a standard NMR relaxation study, NMR relaxation data is usually measured only at one concentration. Study of protein self-association requires sets of data measured at multiple concentrations. A special feature of NMRdyn is the ability to handle multiple sets of NMR relaxation data at different protein concentrations. Using the experimental data, the internal motional parameters for a given residue can be optimized according to a selected model. For residue *i*, this is often achieved by minimizing an error function, as defined in equation 14.
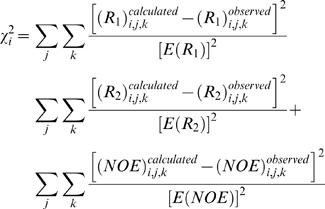
(14)Here, *j* and *k* iterate over the concentrations and frequencies of the measured data, respectively.

Since there are eight basic models in a ‘model-free’ analysis, with different numbers of microdynamic parameters in each model, one has to balance precision with bias in selecting the best dynamic model describing the behavior of a given residue [14]. Akaike's Information Criteria (AIC) [17] has been shown to be a highly reliable way of choosing the best model by selecting the model with the minimum AIC score [14]. For residue *i*, the AIC score for model *m* is given by equation 15 [14], [17].
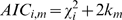
(15)where *k_m_* is the number of parameters in the model *m*. Model selection, based on AIC, has been implemented in NMRdyn.

It should be noted that we have so far discussed NMR relaxation in terms of isotropic diffusion, which applies broadly to the main intended applications, and is an assumption made in earlier studies [13]. However, NMRdyn can also deal with anisotropic motion that may for example result from the formation of an anisotropic oligomer. In many such cases, the oligomer can be described as an axially symmetric ellipsoid, requiring only slight modifications of the spectral density function [18]. To provide an example of the required modifications for the case of anisotropic overall motion, the spectral density function in equation 4 would become:
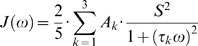
(16)


In this equation, the principal components of the axially symmetric diffusion tensor are *D_x_* = *D_y_* = *D*_⊥_ and *D_z_* = *D*_∥_, and the time constants are *τ*_1_ = (6*D*_⊥_)^−1^, *τ*_2_ = (*D*_∥_+5*D*_⊥_)^−1^, *τ*_3_ = (4*D*_∥_+2*D*_⊥_)^−1^. The coefficients *A_k_* depend on the specific orientation of the respective XH bond vector. In NMRdyn, we adopt a ‘random oligomer’ approach, which assumes that the nature of the monomer-oligomer exchange is fast and non-specific, and is based on the ‘random dimer’ approach of Fushman et al. [12] that was used to analyze the anisotropy of the dimeric state of the dynamin plackstrin homology domain in solution. In this approach, the coefficients *A_k_* can be replaced with their averaged values. For example, in the case of a dimer formed from two spherical monomers joined side-to-side, the values *A_1_* = *1/5*, *A_2_* = *2/5* and *A_3_* = *2/5* are used [12].

### Algorithm and Implementation

**Figure 1:**
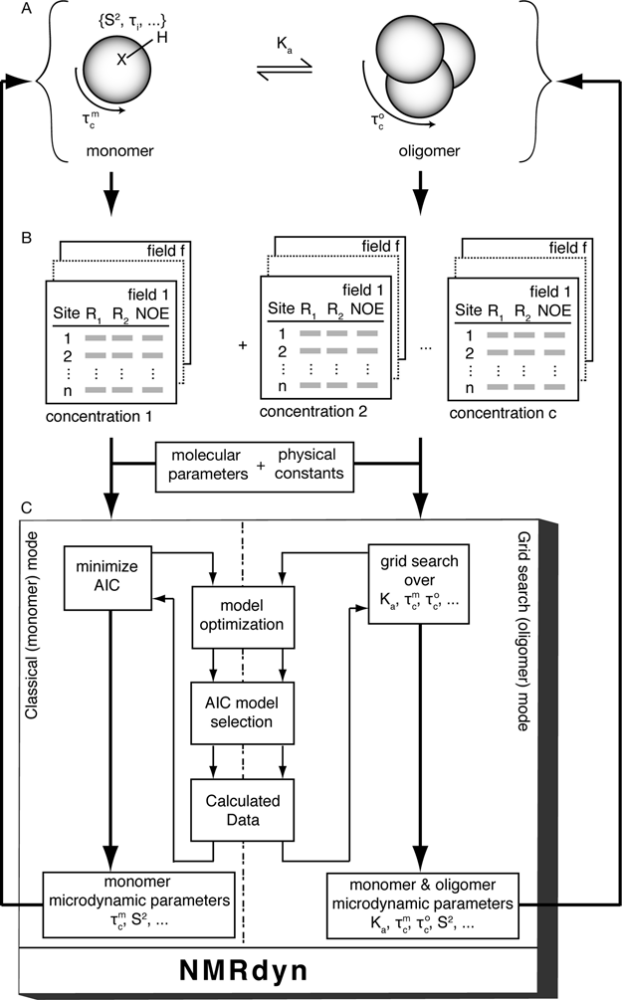
Flowchart describing the operation of NMRdyn. Panel A shows that the system being studied may be a monomeric protein or may include oligomers. The types of parameters that describe the dynamics of the system are labeled. In the monomeric case, relaxation data are usually measured at different magnetic field strengths and at one concentration, for a series of n nuclei (typically the backbone amide nitrogen for each amino acid, or backbone/sidechain ^13^C labeled sites) as shown in panel B. To study self-association, data at multiple concentrations are required. Relaxation data, molecular parameters, and physical constants are used as input into NMRdyn. Panel C (left side) shows that in the case of a monomeric protein, NMRdyn performs a ‘classical’ analysis, where the AIC value is minimized until it and all microdynamic parameters converge with model optimization and model selection performed at each minimization step. For studies of protein self-association (Panel C, right side), a grid-search approach can be applied, resulting in a set of microdynamic parameters describing the monomeric protein and the oligomer.

The core functionality of NMRdyn is the ability to perform a standard model-free analysis of experimental data from ^13^C or ^15^N relaxation experiments to provide motional parameters describing the molecular dynamics of a protein. NMRdyn uses an iterative approach to derive the optimal *τ_c_* for a protein of interest and selects the model along with a set of motional parameters for each residue that best fits the relaxation data. The process of model parameter optimization and model selection forms the basis for the extraction of self-association information from relaxation data, which is achieved using a grid search approach. A flowchart of describing the operation of NMRdyn is shown in Figure 1.

Parameter optimization in NMRdyn is iterative in nature, and stops when pre-defined convergence criteria have been met. Each iteration initiates a nested simplex search strategy. The approach is a non-derivative-based optimization method, which uses a non-degenerate simplex to guide its function sampling. In NMRdyn, a global simplex search is employed to optimize the AIC value, while at each exploratory step in the global simplex search, the selected models are first fitted to the relaxation data for each residue using separate simplex search instances, then tested for validity [19], and finally selected based on their AIC score. To derive protein self-association related parameters, the complete model parameter optimization and model selection procedure is essentially repeated for each grid point in a grid search.

NMRdyn is written in object-orientated C++. Object-orientated programming promotes reusability and extensibility of the code. At the top-level the code is organized into two modules, one dealing with the relaxation analysis algorithm and the other with the implementation of the general user interface. Publicly available libraries were employed in the program. Simplex routines from the publicly available GNU scientific library (GSL) were used to implement optimization routines of the motional parameters. The Qt libraries from Trolltech were used to program the general user interface (GUI). Although NMRdyn was initially developed on a linux operating system, both the GSL and Qt libraries are cross-platform, meaning that NMRdyn can be compiled and run on multiple architectures.

## Results

### Description and Usage

Although NMRdyn can be run via the command line or incorporated into a script to expand the core functionality, the program also presents a user-friendly graphical interface to facilitate analysis. Such an interface is important because it helps the user visualize the motional parameters and relaxation data, which are often large and complex. For a given protein, data for each residue can be measured at multiple field strengths and multiple concentrations, creating an abundance of data. The main design principle of the interface was to allow the user to easily modify the parameters and data, analyze the data, and interpret the results.

**Figure 2:**
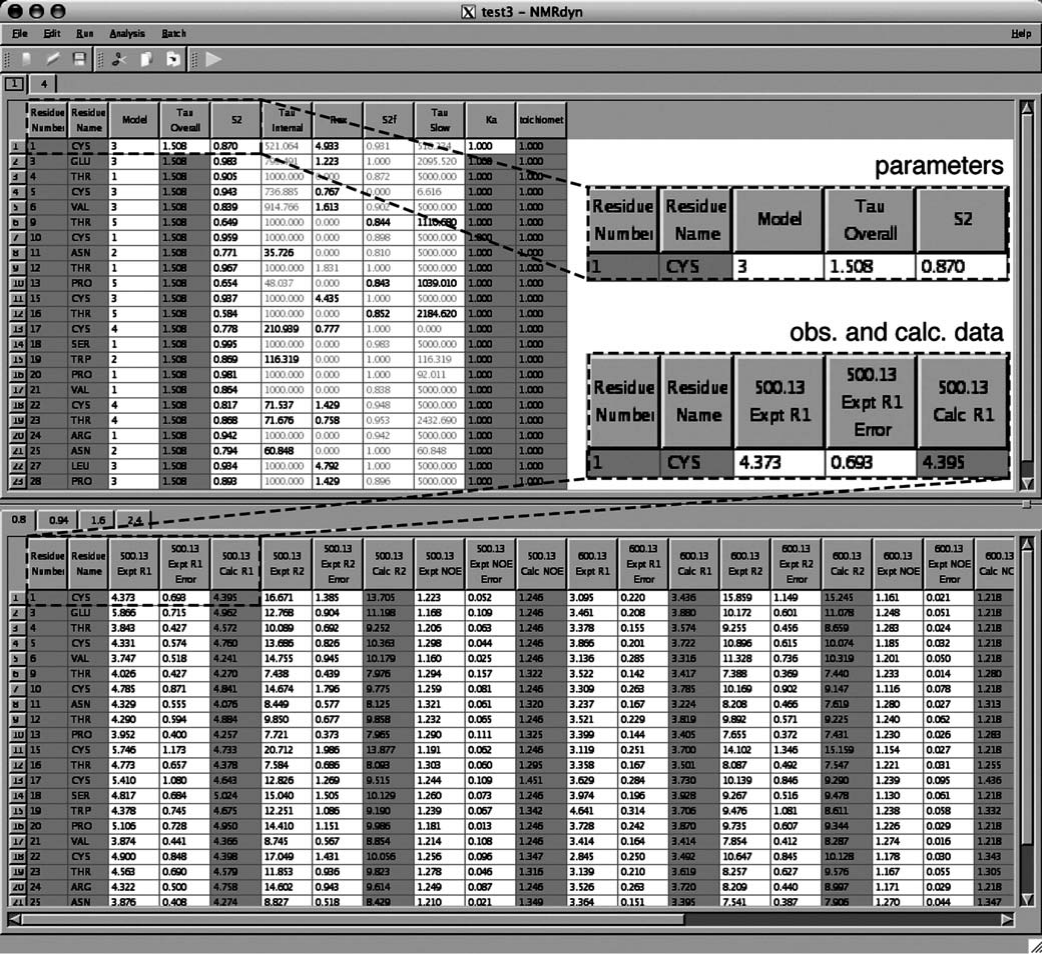
The main interface of NMRdyn. The interface is split into two sections. The top section displays the motional microdynamic parameters and the lower section displays both the observed and calculated data from the relaxation analysis. Both sections are displayed as worksheets so that individual data cells can be easily edited. Above the parameter section is a menu bar, which provides convenient access to the core functions of NMRdyn, including an iterative search to identify the best overall correlation time and set of models for the given relaxation data.

As shown in Figure 2, the interface is split into two sections – one to display the motional parameters (parameter section) and another to display the experimental and calculated data (data section). Each section comprises a number of spreadsheets, allowing the user to easily modify input information. When the user changes the motional parameters in the parameter section, the program dynamically calculates the theoretical data and highlights regions in the calculated data that show significant deviation from the experimental data. This allows the user to interactively determine the effects of the motional parameters.

NMRdyn can automatically optimize the microdynamic parameters for a set of experimental data in a routine relaxation analysis using an iterative protocol or study protein self-association using a grid-search approach. An iterative search can be started after the user has defined project-specific parameters (*i.e.* the number of residues in the protein) and entered the experimental data in the data section. The optimized parameters and selected models from the analysis are displayed in the parameter section, while the calculated data are updated in the data section. To perform a grid-search, the user first specifies the desired range of values for the parameters involved in the grid search, and then the program searches for the optimal values while reporting a summary of the results for each point in the grid. The results for each grid point can be opened as a separate NMRdyn project so that the selected models and optimized parameters can be examined in more detail.

## Discussion

### Practical Applications

**Figure 3:**
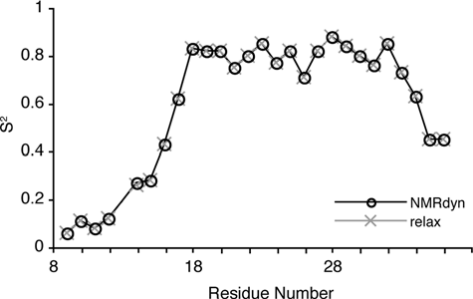
Comparison of order parameters (*S^2^*) computed by NMRdyn (open circles) and relax [5] (crosses) for a relaxation analysis on micelle-bound neuropeptide Y [8]. The original set of relaxation data used was downloaded from the Biological Magnetic Resonance Bank [20]. *S^2^* describes the flexibility of a given residue, with a value close to 1 indicative of high local order, and is one of the most informative parameters from a relaxation analysis. The agreement between the results from NMRdyn and relax validates NMRdyn's implementation.

Understanding the dynamics of a protein is often key to understanding its biological function. Some example applications of NMRdyn are reported in this section. One of the most informative motional parameters is *S^2^*, which describes the internal flexibility of a given amino acid in a protein. In Figure 3, we show the *S^2^* values resulting from a relaxation analysis on a neuropeptide Y dataset [8], assuming isotropic tumbling and a monomeric species, and compare it to the output from relax [5], the most recent program for analyzing NMR relaxation data. The excellent agreement between NMRdyn and relax for both experimental and simulated relaxation data was used to validate NMRdyn's implementation.

**Figure 4:**
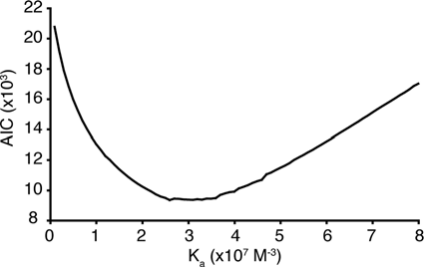
Fixed stoichiometry analysis of kalata B1, the prototypical cyclotide, assuming a monomer-tetramer equilibrium. NMRdyn was used to perform a search over different association constants. The overall Akaike's Information Criteria (AIC) score was used to judge the goodness of the fit, with the aim of obtaining the minimum AIC score. The results indicate that an association constant of approximately 3×10^7^ M^−3^ can be used to describe the formation of the kalata B1 tetramer in solution.

The grid-search function in NMRdyn can be used to study protein self-association. For example, a floating-stoichiometry analysis can be performed to determine the form of the aggregate (*i.e.* whether the oligomer is a dimer, tetramer, etc.) by searching over a grid with various values for the association constant, K_a_, and the size of the oligomer, which can be manipulated in NMRdyn through an interactive input panel. Floating-stoichiometry analysis has been used previously to determine the stoichiometry of oligomers, *e.g.* a floating-stoichiometry analysis of tyrosine phosphatase suggested that it can form tetramers in solution [13]. In the case of the prototypical cyclotide, kalata B1, where the stoichiometry of the oligomer is known to be a tetramer from analytical ultra-centrifugation [11], the value of K_a_ can be refined by searching over different values for K_a_ and selecting the value that gives the best fit of the experimental data as illustrated in Figure 4.

### Conclusion

NMRdyn is a new interactive tool for the analysis of NMR relaxation data. Apart from providing the standard functions to optimize motional parameters according to the ‘model-free’ approach, NMRdyn also features tools to handle protein self-association. NMRdyn is available from http://research1t.imb.uq.edu.au/nmr/NMRdyn.
